# MYLK4 promotes tumor progression through the activation of epidermal growth factor receptor signaling in osteosarcoma

**DOI:** 10.1186/s13046-021-01965-z

**Published:** 2021-05-12

**Authors:** Mengkai Yang, Tao Zhang, Yangfeng Zhang, Xiaojun Ma, Jing Han, Ke Zeng, Yafei Jiang, Zongyi Wang, Zhuoying Wang, Jing Xu, Yingqi Hua, Zhengdong Cai, Wei Sun

**Affiliations:** Department of Orthopedics, Shanghai Bone Tumor Institution, Shanghai General Hospital, Shanghai Jiao Tong University School of Medicine, Shanghai, 200080 P. R. China

**Keywords:** MYLK4, Osteosarcoma, Epidermal growth factor receptor, Growth, Metastasis

## Abstract

**Background:**

Osteosarcoma (OS) is the most common primary bone cancer in adolescents and lung metastasis is the leading cause of death in patients with OS. However, the molecular mechanisms that promote OS growth and metastasis remain unknown.

**Methods:**

We investigated the expression of myosin light chain kinase family members between metastasis and non-metastasis patients in the TARGET database and ensured that only myosin light chain kinase family member 4 (MYLK4) had higher expression in metastatic osteosarcoma patients. Then we confirmed the results by immunohistochemistry (IHC) and Western blotting (WB) of OS tissues. The effect of MYLK4 on the metastasis and proliferation of OS cells was investigated by wound healing, Transwell and colony-formation assays. Mass spectrum analysis was used to ensure the new binding protein of MYLK4. Tissue microarrays analysis was used to show the correlation between MYLK4 and pEGFR (Y1068). A series of in vivo experiments were conducted to reveal the mechanisms by which MYLK4 modulated the metastasis and proliferation of OS.

**Results:**

Myosin Light Chain Kinase Family Member 4 (MYLK4) was significantly upregulated in metastatic human OS tissues. Growth and metastasis of OS could be accelerated by MYLK4 overexpression, whereas silencing MYLK4 expression resulted in decreased cell growth and metastasis. Mechanistically, mass spectrum analysis showed that MYLK4 interacted with the epidermal growth factor receptor (EGFR) in osteosarcoma cells and promoted growth and metastasis via the EGFR signaling pathway. Tissue microarrays analysis also showed that MYLK4 expression had a positive correlation with the expression of pEGFR (Y1068). Moreover, the EGFR inhibitor gefitinib could partially reverse the effect of cell proliferation and metastasis caused by MYLK4 overexpression. Importantly, the combination of MYLK4 and EGFR inhibitors had synergistic effects on growth and metastasis of OS in vitro and in vivo.

**Conclusion:**

Our results indicate that MYLK4 promotes OS growth and metastasis by activating the EGFR signaling pathway and can be a novel therapeutic target for the treatment of OS patients.

**Supplementary Information:**

The online version contains supplementary material available at 10.1186/s13046-021-01965-z.

## Background

Osteosarcoma (OS) is the most common bone tumor and is often found in adolescents [1]. Primary OS has a five-year survival rate of 69%, but when the tumor metastasizes, the survival rate is only 15–30% [2]. It has been reported that 20% of patients with OS have a distant metastasis when they are first diagnosed [3]. Among the numerous causes of death in patients with OS, lung metastasis causes the highest mortality. Thus, it is necessary to explore the underlying molecular mechanisms in distant metastasis of OS.

Myosin light chain kinase (MYLK) was discovered approximately 40 years ago as a skeletal and smooth muscle enzyme [4]. Since then, knowledge regarding MYLK has gradually increased. The MYLK family is made up of four different kinases (MYLK, MYLK2, MYLK3, and MYLK4) that are encoded by different genes [5]. Numerous cell activities, such as cell migration, invasion, and proliferation, are influenced by MYLK [6]. Dynamic changes in the cytoskeleton are necessary for invasion and metastasis potential of cancer cells. Cytoskeletal myosin II phosphorylated by MYLK increases the metastatic potential of tumor cells, and MYLK-dependent cytoskeleton rearrangement adjusts the angiogenesis of the vascular endothelial barrier, which is an inevitable step in cancer metastasis [7]. Bao et al. found through bioinformatics analysis that MYLK2 was associated with poor prognosis in triple-negative breast cancer [8]. For those with hepatocellular carcinoma, Wang et al. indicated that MYLK2 was upregulated in tumor tissues and was linked with a lower survival rate [9]. However, studies on MYLK3 and MYLK4 are limited. MYLK3 has an important role in preventing cardiomyocyte atrophy [10]. Moreover, in patients with heart failure, MYLK4 has a vital role in the altered cytoskeletal network of cadiomyocytes [11]. Lee et al. found that MYLK4 was a monoallelically expressed gene through high-density single nucleotide polymorphism chips that have an important role in the development of colorectal cancer cells [12]. Although MYLK family members were found to be associated with the development of tumors in previous studies, how they are involved in the growth and metastasis of OS remains unclear.

Cell surface receptors that can be stimulated by various growth factors can promote the proliferation and invasion of multiple cancers. As transmembrane protein receptors, receptor tyrosine kinases such as the epidermal growth factor receptor (EGFR) have an important effect on cell physiology [13]. EGFR overexpression is generally found in many types of human cancer and is currently used in multiple cancer treatment targets in clinical practice [14]. A poor prognosis in those with certain cancers such as cervical, bladder, head and neck, and ovarian cancers, has been found to have an important correlation with EGFR overexpression [15]. In patients with OS, high EGFR expression is also associated with high proliferation activity and metastasis potential, as well as poor prognosis [16]. In addition, dysfunction of signaling in several downstream EGFR pathways such as PI3K/AKT and JAK/STAT3 has also been implicated in OS development and metastasis [17, 18]. Recently, numerous EGFR antibodies and inhibitors have shown substantial application potential in tumor therapy [19]. In one clinical trial, EGFR inhibitors were shown to have good therapeutic effect for those with OS [20]. Whether the EGFR signaling pathway could be affected by MYLK4 in those with OS needs to be further investigated.

In the present study, we found that MYLK4 interacted with EGFR. This interaction promoted OS progression through the EGFR signaling pathway in vivo and in vitro. MYLK4 overexpression led to EGFR phosphorylation, whereas MYLK4 knockdown reduced EGFR phosphorylation. More importantly, we demonstrated that the combination of MYLK4 and EGFR inhibitors exhibits a promising synergistic effect for OS treatment. In general, this study demonstrated that MYLK4 promotes OS proliferation and metastasis via the activation of the EGFR signaling pathway.

## Materials and methods

### Database analysis

We downloaded the mRNA expression data of patients with OS from the genomic data commons data portal (https://portal.gdc.cancer.gov/). Complete clinical information of the above patients were downloaded from the TARGET database (https://ocg.cancer.gov/programs/target). There were 85 patients included in this database. The clinical information in the TARGET datasets were shown in Supplementary table 1. The univariate cox regression analysis was used to assess the correlation between gene expressions and prognosis of OS patients. The mRNA expression data of OS samples in GSE12865 which contained two normal samples were downloaded from the NCBI gene expression omnibus (https://www.ncbi.nlm.nih.gov/geo/, GSE12865).

### Survival analyses

We compared the overall survival of OS patients between high-MYLK4 group and low-MYLK4 group. We performed the log-rank test to verify if the differences in survival time were significant. Kaplan-Meier curves were used to visualize the survival of different groups and the differences among them.

### Cells and cell culture

The Cell lines 143B (human OS cells), U2OS (human OS cells), SAOS2 (human OS cells), HOS (human OS cells), MG63 (human OS cells), SJSA-1(human OS cells) and HEK-293 T cell lines were obtained from ATCC (Manassas, VA, USA). MG63.2 cell was derived from the metastasis of parental MG63, as previously reported [21]. All cell lines were maintained in DMEM supplemented with 10% FBS and 1% penicillin/streptomycin in a humidified incubator at 37 °C and 5% CO_2_. These cell lines were mycoplasma-free and routinely authenticated by quality examinations of morphology and growth profile.

### Cell transfection

The plasmids coding for human MYLK4, EGFR and relevant empty plasmid were obtained from Youbio Biological Technology Co., Ltd. (Changsha, China). The MYLK4 lentiviral shRNA plasmid was supplied by Genomeditech (Shanghai, China). The sequences targeting MYLK4 were as follows: shMYLK4–1, CCGGATCGATGAGAGCTACAATTTGCTCGAGCAAATTGTAGCTCTCATCGATTTTTTG, shMYLK4–2, CCGGCATCGCCTATATGCTACTTAGCTCGAGCTAAGTAGCATATAGGCGATGTTTTTG.

The lentivirus was acquired in HEK293T cells. We co-transfected indicated plasmids and helper virus packaging plasmids pMD2.G and psPAX2 by using Lipofectamine 3000 (Invitrogen, Carlsbad, CA, USA) in HEK293T cells. Following infection for 48 h, supernatants containing lentivirus were harvested.

### Immunofluorescence (IF) staining

All the cells were fixed in 4% paraformaldehyde, permeabilized with 0.5% Triton X-100 and incubated with Alexa Fluor 594 phalloidin for 90 min (Abcam, MA, USA). Then, the cells were incubated with DAPI for 10 min (Beyotime, Shanghai, China). Immunofluorescence images were obtained by using fluorescent microscope (Leica, Germany).

### Cell proliferation and colony-formation assays

Cell proliferation was estimated by the Cell Counting Kit-8 (CCK8) (Dojindo, Tokyo, Japan) based on the manufacturer’s instructions. For colony-formation assays, cells were plated in six-well plates at a density of 500 cells per well and incubated at 37 °C for two weeks until the cells grew to visible colonies. Then, the medium was discarded and the remaining cells were washed with PBS for three times. Colonies were fixed with 4% paraformaldehyde and stained with 0.1% crystal violet for 15 min.

### Transwell invasion assay

Cell invasion capabilities were detected by using Transwell chambers precoated with the Matrigel matrix as previously described [22]. Cells were seeded into the upper compartments at a density of 5 × 10^4^ cells/well in 100 μl serum-free media and the lower chamber were added 600 μl DMEM with 10% fetal bovine serum as a chemoattractant. After which the plates were incubated for 12 h at 37 °C. The non-invaded cells were mildly removed from the upper chamber using a cotton swab and the invaded cells on the bottom surface were fixed with 4% paraformaldehyde and stained with 0.1% crystal violet. Images were acquired by an inverted microscope (Olympus), and the invaded cells were counted manually.

### Wound-healing migration assay

Wound-healing migration assay was performed as previously described [22]. Cells were seeded in six-well plates and grew until they reached full confluence. Use a sterile 100 μL pipette tip to create a “wound”. Observe the breadth of “wound” at different time points with a fluorescence microscope (Olympus). The distances were measured by ImageJ software (National Institutes of Health, MD).

### Co-immunoprecipitation assay

Co-immunoprecipitation (Co-IP) assay was performed using 143B and HEK-293 T cells. The protein lysate was extracted by NP40 (Beyotime, P0013F) for 30 min on ice, and centrifuged at 12,000×g for 20 min. Every 1 μg of the primary antibody or IgG were mixed with 500 μg total proteins and then were shaken for 1 h at 4 °C. At last, the Protein G beads (Santa Cruz Biotechnology, USA) were added to the mixture and shaken at 4 °C overnight. The beads were then washed 3 times using immunoprecipitation buffer. Loading buffer (2x) was mixed with the beads and boiled for 15 min. Then the samples were used for western blot analysis.

### Western blotting

Cells and tumor tissues were lysed with RIPA buffer (Beyotime, Shanghai, China) which was mixed in advance with a protease and a phosphatase inhibitor cocktail (Sigma-Aldrich, USA) for 30 min on ice. After centrifugation at 12,000×g for 20 min at 4 °C, the supernatants were collected and quantified using BCA assay (Beyotime, Shanghai, China). The 40–60 μg of each sample was resolved on SDS-PAGE and transferred to polyvinylidene difluoride (PVDF) membranes through Bio-Rad (Hercules, CA, USA). These membranes were incubated with 5% non-fat milk dissolved by TBST (TBS and 0.1% Tween20) for 1 h at room temperature and incubated overnight at 4 °C with primary antibodies. The next day, membranes were washed with TBST for three times every ten minutes and then incubated with secondary antibody (Sigma-Aldrich). After washing three times, signals were detected through ECL detection reagents. Primary antibodies: P-EGFR (Tyr1068) (#3777, Cell Signaling), EGFR (#4267, Cell Signaling), P-AKT (#4060, Cell Signaling), AKT (#4685, Cell Signaling), P-ERK (#4370, Cell Signaling), ERK (#4695, Cell Signaling), MYLK4 (24309–1-AP, Proteintech).

### Mass spectrometry analysis

The analysis was performed in an HPLC system (Easy nLC1000, Thermo Fsher Scientific, USA) and mass spectrometer (Orbitrap Elite, Thermo Fisher Scientific, USA). The IP beads were collected from a 10 cm dish of Flag-MYLK4 overexpression 143B cells. The samples were subjected to western blot and then stained by silver staining.

### Immunohistochemistry (IHC)

IHC assays were conducted as reported previously [22]. OS tissue array was stained with anti-MYLK4 antibody and anti-P-EGFR (Y1068) antibody. Images were obtained with Leica microscope (Leica, DM4000b). The immunohistochemical scores were calculated by formula as followed. H-SCORE = ∑(PI×I) = (percentage of cells of weak intensity *1) + (percentage of cells of moderate intensity *2) + (percentage of cells of strong intensity *3).

### His-tag pull-down assay

GST-MYLK4 and His-EGFR (668–1210) were expressed in bacteria and purified in accordance with the manufacturer’s instructions. Then, 10 μg of His-EGFR was mixed with 10 μg of GST-MYLK4 or GST and incubated with Ni-NTA agarose, for the His-tag pull-down assay. The pellets were washed three times and boiled in SDS buffer for 10 min. At last, we examined the bound proteins by western blot with anti-GST and anti-His tag.

### In vitro kinase assay

For in vitro kinase assays, GST-MYLK4 purified protein was incubated with His-EGFR (668–1210) purified protein at 30 °C in a total volume of 30 ml of kinase assay buffer for 1 h. The reaction mixtures were stopped by SDS buffer and analyzed by Western blots using pIMAGO for phosphoprotein detection on western blot kit (TYMORA, 800–10).

### In vivo animal experiments

The animal experiments were approved by the Animal Research Committee of the Shanghai General Hospital (Ethical code: 2019AW004) and were performed in accordance with established guidelines. For the growth and metastasis assays in vivo, four-week-old male nude mice were orthotopically inoculated into the right tibia with a micro syringe. A total of 1 × 10^6^ corresponding cells suspended in 20 μL of PBS were injected into each nude mouse. One month later, the mice were sacrificed, the tumors and individual lung tissues were excised and fixed with 4% paraformaldehyde. Metastatic lung tissues were analyzed by H&E staining. For verification of the effect on combination of MYLK4 inhibitor and EGFR inhibitor in vivo, four-week-old male nude mice were orthotopically inoculated into the right tibia with a micro syringe, too. 143B cells were harvested and washed three times with cold PBS. Then, total of 1 × 10^6^ cells suspended in 20 μL of PBS were injected into the medullary cavity of the tibia. When the tumors in the tibia were macroscopically visible, the mice were randomly divided into four groups: control group, ML-7, Gefitinib single treated group and ML-7 plus Gefitinib treated group and respectively received i.p. injection of gefitinib (50 mg/kg per 2 days), ML-7 (1 mg/kg per 2 days), the combination of gefitinib (50 mg/kg per 2 days) and ML-7 (1 mg/kg per 2 days) as compared with mice injected with DMSO (control group). One month later, all mice were killed, the tumors and individual lung tissues were excised and fixed with 4% paraformaldehyde. Metastatic lung tissues were analyzed by H&E staining.

### Hematoxylin and eosin staining

Lung tissues were fixed overnight, embedded in paraffin and then cut into serial sections (4-μm thick). The sections were subjected to hematoxylin and eosin (H&E) staining. Representative images were acquired using a microscope (Leica).

### Statistical analysis

All data are presented as mean ± standard derivation (S.D.). The Student t test was used for the comparison of measurable variants of two groups (*P* < 0.05 was considered significant) unless otherwise indicated. All experiments were performed at least three times except for the experiments using the animal models. The variance per assay was similar between the groups statistically compared. No data points in our study were excluded.

## Results

### MYLK4 is upregulated in metastatic OS tissues

**Fig. 1:**
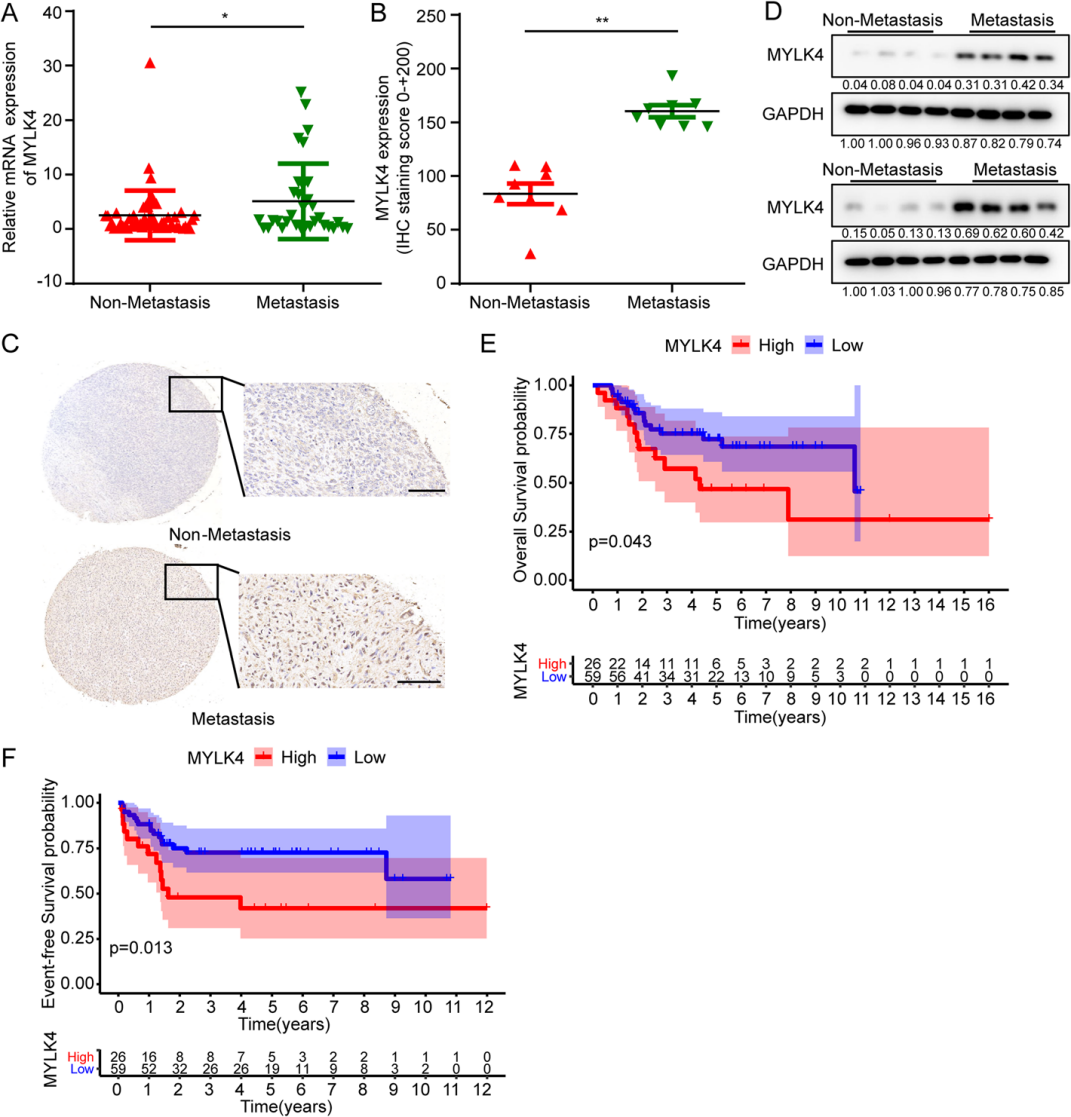
MYLK4 is upregulated in metastatic human OS tissues. **a** The expression of MYLK4 in non-metastasis and metastasis OS samples in TARGET database. Statistically significant differences (t-test), *P < 0.05. **b** Immunohistochemistry analyses of MYLK4 expression levels in 8 paired metastatic human OS and non-metastatic human OS tissues. Statistically significant differences (t-test), **P < 0.01. **c** Representative immunohistochemical assays of MYLK4 expression in metastatic human OS (down) and non-metastatic human OS tissues (up). **d** Western blot analysis of MYLK4 expression in metastatic human OS compared with non-metastatic human OS tissues. **e-f** KM survival curves for overall survival and event-free survival of osteosarcoma patients from MYLK4-high and MYLK4-low groups

In order to illuminate the function and clinical importance of myosin light chain kinase family members (MYLK, MYLK2, MYLK3 and MYLK4) in OS, we first analyzed MYLK, MYLK2, MYLK3, and MYLK4 expression using the Therapeutically Applicable Research to Generate Effective Treatments database to understand the function and clinical importance of the MYLK family. We found that only MYLK4 had a higher expression in patients with OS during metastasis (*P* = 0.0415) (Fig. 1a; Supplementary Fig. 1A-C). Furthermore, we used an immunohistochemistry assay to determine whether MYLK4 expression is related to OS metastasis. We found that MYLK4 expression was higher in metastatic OS tissues than that in non-metastatic tissues (Fig. 1b and c). The clinical characteristics of the osteosarcoma patients in immunohistochemistry assay were shown in Supplementary table 2. Additionally, we examined MYLK4 protein levels in these samples through Western blot analysis and found that metastatic OS tissues had higher MYLK4 protein levels than that in the non-metastatic groups (Fig. 1d). Furthermore, using data from the patient cohort obtained in the database, the Kaplan-Meier (KM) analysis showed that patients with high expression of MYLK4 had lower overall survival rates and event-free survival rates than patients with low expression of MYLK4 (Fig. 1e and f). The survival time of the osteosarcoma patients in TARGET database was shown in Supplementary table 3. We also found that only MYLK4 had higher expression in OS tissues than that in normal human osteoblasts in GSE12865 (Supplementary Fig. 1D-G), and we verified that MYLK4 was generally expressed in OS cell lines (Supplementary Fig. 1H). We also found that U2OS, Saos2 and MG63 had lower expressions than SJSA-1, HOS, MG63.2 and 143B. This result was consistent with the characteristics of human osteosarcoma cell lines. U2OS, Saos2 and MG63 had lower invasion capacity than SJSA-1, HOS, MG63.2 and 143B. These results demonstrate that MYLK4 shows high expressions in metastasis OS samples and can be a risk factor in OS patients.

### MYLK4 promotes OS metastasis

**Fig. 2:**
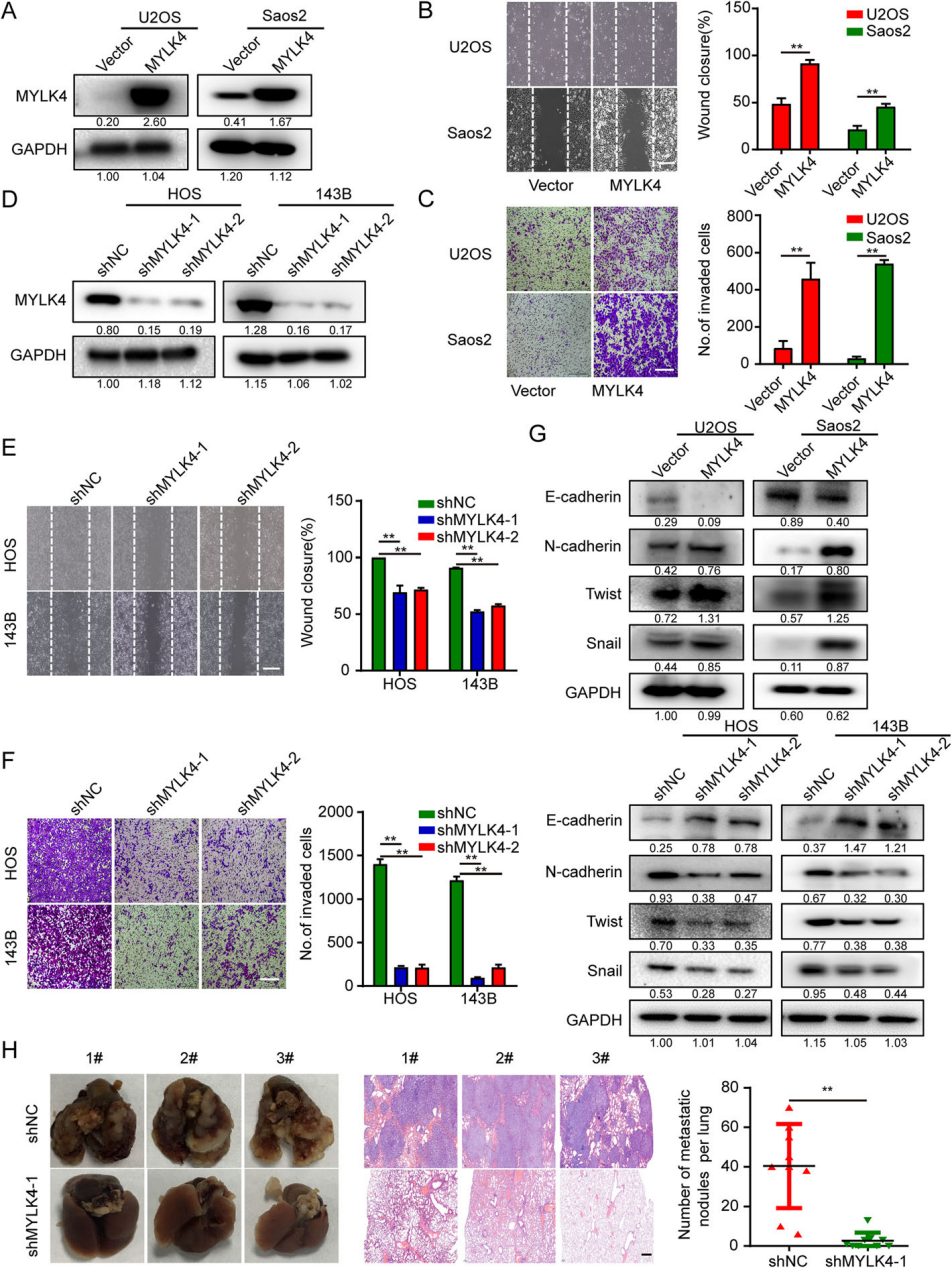
MYLK4 promotes osteosarcoma cell metastasis in vivo and in vitro. **a** The MYLK4 overexpression effects were confirmed by western blotting. **b** The effect of MYLK4 overexpression on OS cell migration was assessed by a wound healing assay. Representative images of migration were shown in the left panel. The degrees to which the wounds healed in the indicated groups were shown in the histogram. The bars indicate the mean ± s.d. Statistically significant differences (t-test), **P < 0.01, Scale bars = 250 μm. **c** The effect of MYLK4 overexpression on OS cell invasion was assessed by a transwell assay (left panel). The relative proportions of invading cells were shown in the histogram. The bars indicate the mean ± s.d. Statistically significant differences (t-test), **P < 0.01, Scale bars = 200 μm. **d** The MYLK4 knockdown effects were confirmed by western blotting. **e** The effect of MYLK4 knockdown on OS cell migration was assessed by a wound healing assay. Representative images of migration were shown in the left panel. The degrees to which the wounds healed in the indicated groups were shown in the histogram. The bars indicate the mean ± s.d. Statistically significant differences (t-test), **P < 0.01, Scale bars = 250 μm. **f** The effect of MYLK4 knockdown on OS cell invasion was assessed by a transwell assay (left panel). The relative proportions of invading cells were shown in the histogram. The bars indicate the mean ± s.d. Statistically significant differences (t-test), **P < 0.01, Scale bars = 200 μm. **g** The expression of EMT markers was detected by western blotting in MYLK4 over-expression and MYLK4-knockdown OS cells. **h** The number of lung metastasis nodules formed by MYLK4-knockdown 143B cells and control cells in orthotopic osteosarcoma implanted mice was shown in the right panel. Statistically significant differences (t-test), **P < 0.01. Representative images of lung morphology and lung metastatic nodules formed by MYLK4-knockdown 143B cells and control cells were shown in the left panel. Corresponding HE staining was shown in the middle panel. Scale bars = 100 μm

Cell migration and invasion is a critical step in tumor metastasis. Lung metastasis is the most common transference process of OS patients, which is the major cause of death in OS patients. The MYLK family members have been proved to have effect on metastasis in many tumors, but the role of MYLK4 in OS is poorly understood. Therefore, we explored the function of MYLK4 on OS cell migration and invasion. In order to investigate the causal role of MYLK4 played in OS progression, we constructed MYLK4 down-regulation HOS and 143B stable cell lines, also MYLK4 overexpression U2OS and Saos2 stable cell lines (Fig. 2a, d). Results of the “wound healing” migration assay indicated that MYLK4 overexpression or knockdown significantly promoted or inhibited cell migration in corresponding OS cells, respectively (Fig. 2b, e). The result of Transwell invasion assay also obtained similar result (Fig. 2c, f). Moreover, we investigated whether the function of MYLK4 in OS cells promoted the epithelial to mesenchymal transition (EMT) process. E-cadherin is the epithelial marker while N-cadherin, Twist and Snail are the mesenchymal marker in EMT [23]. As shown in Fig. 2g, MYLK4 knockdown suppressed N-cadherin and Twist and Snail expression and increased E-cadherin expression. By contrast, MYLK4 overexpression increased N-cadherin and Twist and Snail expression and suppressed E-cadherin suppression. Furthermore, we found that TGF-β can be induce MYLK4 expression in OS cells (Supplementary Fig. 2A). Meanwhile, MYLK4 knockdown slowed down the induction EMT effect of TGF-β in OS cells (Supplementary Fig. 2B). In addition, we detected the effect of MYLK4 on cytoskeletal organization of the OS cell. The F-actin stress fibers were observed by Alexa Fluor 594 phalloidin. The result showed that the number of F-actin stress fibers in MYLK knockdown cells were reduced in Supplementary Fig. 3. Next, we further verified the role of MYLK4 in OS metastasis in vivo. Because the two shRNA have almost the same efficiency (Fig. 2d), we choose shRNA-1 for animal experiment. The numbers of lung metastatic nodules in MYLK4-knockdown orthotopic OS-implanted mice were fewer than those in the control group (Fig. 2h). Taken together, these findings suggest that MYLK4 could promote OS metastasis by regulating cell migration, invasion, and EMT process.

### MYLK4 increases OS cell proliferation

**Fig. 3:**
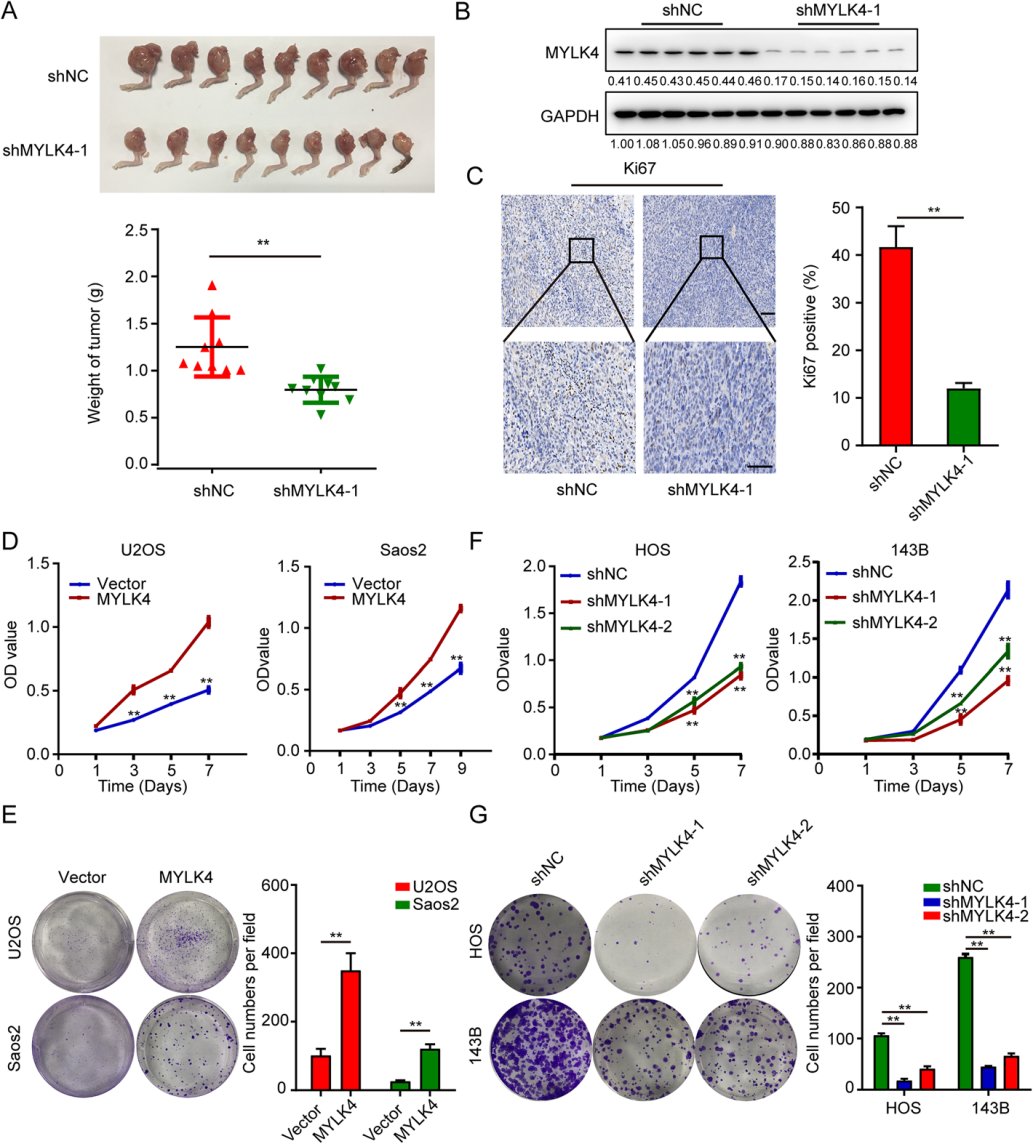
MYLK4 promotes osteosarcoma cell proliferation. **a** Osteosarcoma tissues from mice with tumor xenografts inoculated with MYLK4-knockdown 143B cells. The weight of osteosarcoma samples was shown in the down panel. Statistically significant differences (t-test), **P < 0.01. **b** Western blotting were performed to detect the shRNA interference efficiency in vivo. **c** The expression of Ki67 was detected by IHC in orthotopic OS implanted-mice tumor tissues formed by MYLK4-knockdown 143B cells and control cells. The results of Ki67 positive (%) were shown in the right panel. The bars indicate the mean ± s.d. Statistically significant differences (t-test), **P < 0.01, Scale bars = 100 μm. **d** The effect of MYLK4 overexpressing on OS cell proliferation was assessed by the CCK8 assay. The bars indicate the mean ± s.d. Statistically significant differences (t-test), **P < 0.01. **e** The effect of MYLK4 overexpression on colony formation in OS cells. Representative images of colony formation were shown in the left panel. The numbers of cells in the indicated groups are shown in the histogram. The bars indicate the mean ± s.d. Statistically significant differences (t-test), **P < 0.01. **f** Cell proliferation was measured by CCK8 assay with/without MYLK4 knockdown in OS cells. The bars indicate the mean ± s.d. Statistically significant differences (t-test), **P < 0.01. **g** The effect of MYLK4 knockdown on colony formation in OS cells. Representative images of colony formation were shown in the left panel. The numbers of cells in the indicated groups were shown in the histogram. The bars indicate the mean ± s.d. Statistically significant differences (t-test), **P < 0.01

According to the result of animal experiment, we also found MYLK4-knockdown orthotopic osteosarcoma implanted mice not only had the reduced lung metastasis, but also had the smaller tumor weight (Fig. 3a). We also detected the efficiency of shRNA in vivo, we found the expression of MYLK4 is significantly reduced in shMYLK4 group (Fig. 3b). Immunohistochemical staining results showed that the percentage of Ki67-positive cells was lower in orthotopic OS implanted-mice due to MYLK4 knockdown in 143B cells (Fig. 3c). Therefore we wonder whether MYLK4 also plays an important role in cell growth and proliferation of OS. Our results demonstrated that MYLK4 overexpression in U2OS and Saos2 stable cell lines accelerated proliferation and colony-formation ability compared to that in the control (Fig. 3d and e). Inversely, MYLK4 knockdown in 143B and HOS stable cell lines inhibited proliferation and colony-formation ability (Fig. 3f and g). Therefore, this results indicate that MYLK4 could also promote OS cell proliferation.

### MYLK4 interacted with EGFR

**Fig. 4:**
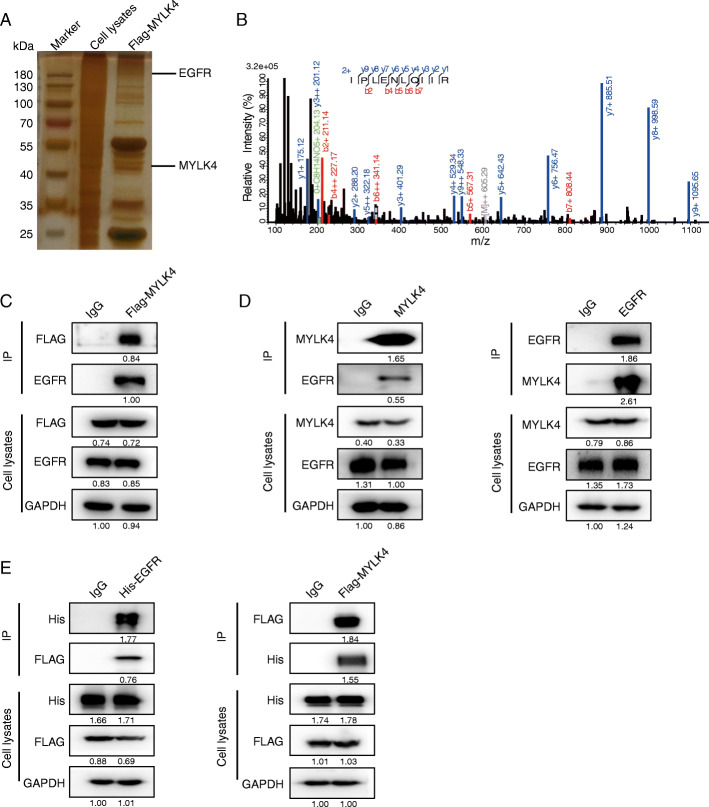
MYLK4 interacts with EGFR. **a** Silver staining of whole cell lysates (WCL) and immunoprecipitates (IP) of FLAG-MYLK4 143B cells. The indicated bands were sequenced by mass spectrometry (MS) analysis. **b** Secondary peptide structure of EGFR acquired by MS. **c** 143B cells were transfected with Flag-tagged MYLK4 construct and the interaction between exogenous MYLK4 and EGFR was detected by IP and immunoblotting (IB). **d** Interaction between endogenous MYLK4 and EGFR by IP analyses in 143B cells. **e** HEK-293 T cells were cotransfected with Flag-tagged MYLK4 and HIS-tagged EGFR constructs, and the interaction between exogenous MYLK4 and EGFR was detected by IP and IB

To further study the underlying mechanism by which MYLK4 promoted OS growth and metastasis, we performed mass spectrometry followed by silver staining in 143B-MYLK4-FLAG cell line and confirmed the EGFR as one of the new potential proteins interacting with MYLK4 based on the mass spectrometry results (Fig. 4a and b) (Supplementary table 4). Moreover, the interaction between MYLK4 and the EGFR was verified based on the results of immunoprecipitation experiments using 143B stably expressing MYLK4-FLAG (Fig. 4c). Furthermore, the presence of the EGFR was detected by the immunoprecipitation of endogenous MYLK4, and the endogenous EGFR was detected in the 143B cell line based on the results of the reciprocal Co-IP assays (Fig. 4d). Reciprocal Co-IP assays also indicated that the exogenous EGFR and MYLK4 interact with each other in 293 T (Fig. 4e). Taken together, these results strongly support that the EGFR is a bona fide binding protein of MYLK4.

### MYLK4 enhances the EGFR signaling pathway in OS

**Fig. 5:**
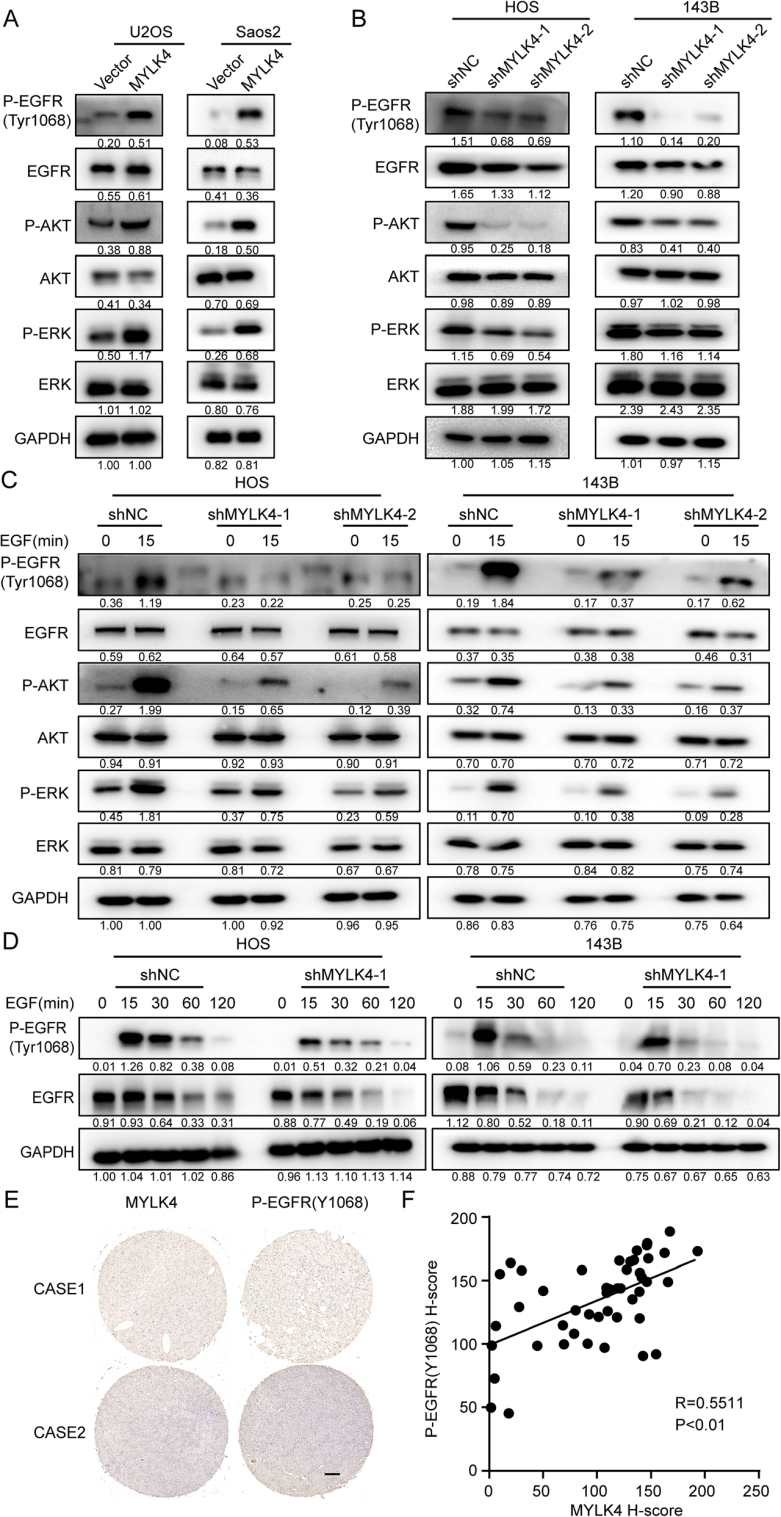
MYLK4 promotes EGFR phosphorylation and the downstream signaling pathway. **a** The expression of p-EGFR, p-AKT and p-ERK was detected by western blotting in MYLK4-overexpressing cells. **b** The expression of p-EGFR, p-AKT and p-ERK was detected by western blotting in MYLK4-knockdown cells. **c** The expressions of p-EGFR, p-AKT and p-ERK were detected by western blotting with/without EGF stimulation for 15 min. **d** The expression of p-EGFR was detected by western blotting with EGF (50 ng/ml) stimulation for different time period respectively in MYLK4-knockdown and the control cells. **e** The expressions of MYLK4 and P-EGFR (Y1068) expression in human OS tissues were detected by IHC assay. Scale bars = 200 μm. **f** The score of MYLK4 and P-EGFR (Y1068)-positive staining were quantified and the Spearman correlation was performed

Since MYLK4 has an interaction with EGFR, we further to find that whether MYLK4 could promote the phosphorylation of EGFR and enhance the EGFR signaling. Our results demonstrated that MYLK4 overexpression in U2OS and Saos2 cell lines promoted the EGFR signal pathway, as evidenced by increased expression of p-EGFR, p-AKT, and p-ERK. Conversely, MYLK4 knockdown restrained EGFR signaling in OS cells (Fig. 5a and b). Next, we detected the changes of EGFR phosphorylation following epidermal growth factor (EGF) stimulation for 15 min between MYLK4-knockdown cells and the control cells and found MYLK4-knockdown cells exhibited EGFR phosphorylation decrease, along with p-AKT and p-ERK downregulation (Fig. 5c). Then we further the changes of EGFR phosphorylation following EGF stimulation for a 120-min time course and also found that knockdown of MYLK4 restrained the activation of the EGFR signaling pathway in HOS and 143B cells treated with EGF (Fig. 5d). In order to investigate the correlation between MYLK4 and EGFR (Y1068) in OS, their expression levels were measured in 47 human OS tissues by Immunohistochemistry (IHC). IHC results showed a positive correlation between the expression of MYLK4 and p-EGFR (Y1068) and the Spearman correlation coefficient was 0.5511 and *p* < 0.01 (Fig. 5e and f). In addition, we treated the 143B-MYLK4-FLAG cell line with or without 25 mM Gefitinib for 24 h. Then the phosphorylated MYLK4 was detected by anti-Phospho-Tyrosine antibody. The result in Supplementary Fig. 4A showed that the expression of p-Tyr of MYLK4 didn’t change after the stimulation of Gefitinib. It meant that EGFR couldn’t phosphorylate MYLK4. Furthermore, we speculated that if MYLK4 could directly phosphorylate EGFR. Thus, we constructed a truncate of EGFR which owned the intracellular tyrosine kinase domain (aa 668–1210). Then, His-tag pull-down assay was carried out to determine whether there is a direct interaction between MYLK4 and EGFR (668–1210) in vitro. MYLK4 and EGFR (668–1210) were bacterially expressed and purified as GST and His-tag fusion proteins, respectively. As shown in Supplementary Fig. 4B, GST-MYLK4, but not GST, bound to His-tagged EGFR, indicating direct binding between these proteins in vitro. Then, we further test whether MYLK4 can phosphorylate EGFR through kinase assay in vitro. As shown in Supplementary Fig. 4C, EGFR was phosphorylated by MYLK4. Collectively, these findings suggest that MYLK4 is associated with p-EGFR (Y1068).

We then tested whether MYLK4-mediated OS migration and invasion was depend on EGFR signaling pathway. Our results showed that EGFR overexpression could partially reverse the inhibition of OS cell migration and invasion caused by MYLK4 knockdown (Supplementary Fig. 5A, C and D). We also found the EGFR inhibitor gefitinib partially reversed the promotion of cell migration and invasion caused by overexpression of MYLK4 (Supplementary Fig. 5B, E and F). Therefore, our results indicated that MYLK4 promoted OS progression through the activation of the EGFR signaling pathway.

### MYLK4 inhibitor ML-7 and EGFR inhibitor gefitinib synergize in suppressing the growth and metastasis of OS in vitro and in vivo

**Fig. 6:**
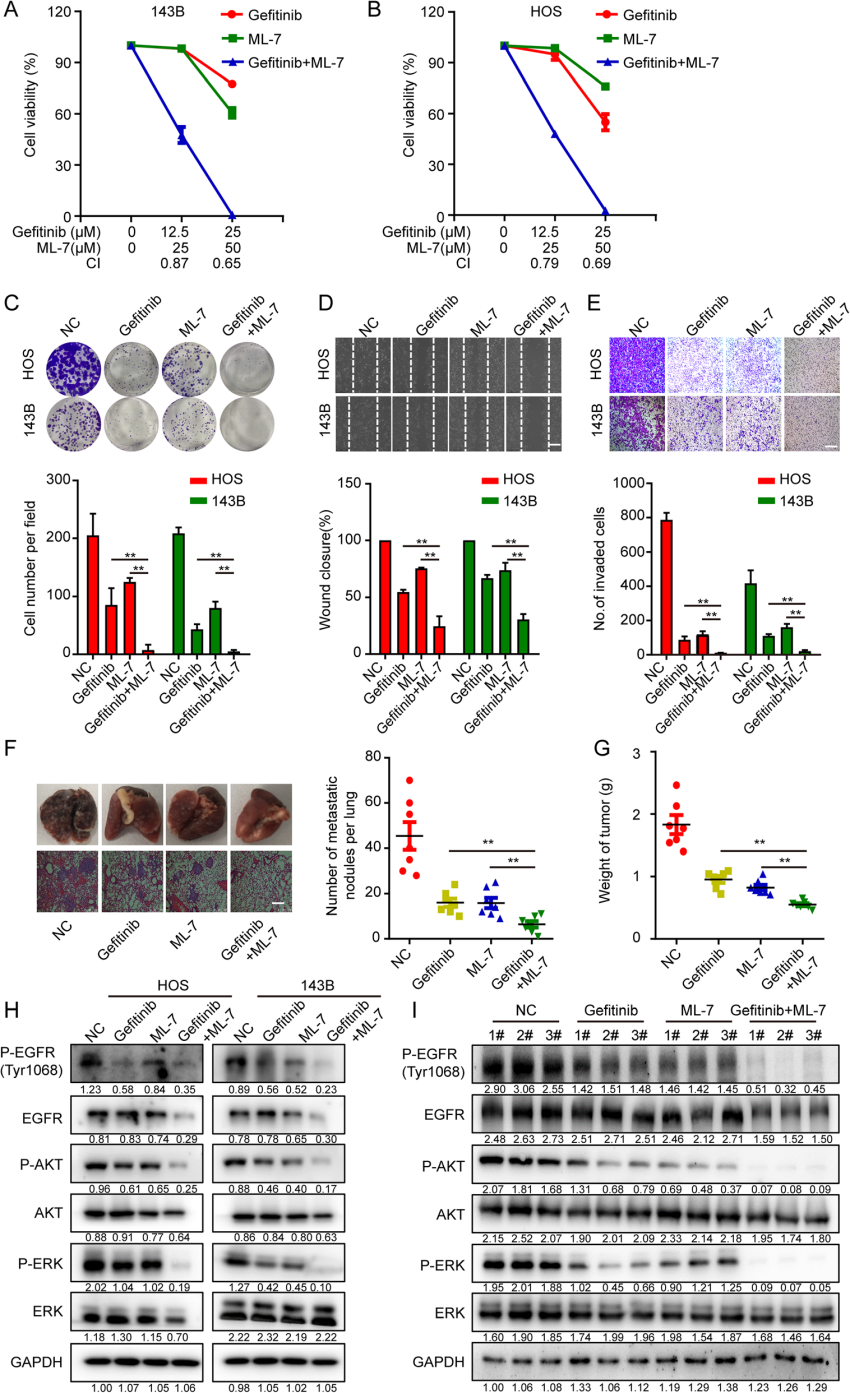
The inhibitor of MYLK4 shows synergistic effect with the EGFR inhibitor in OS in vitro and in vivo. **a** 143B and **b** HOS cells treated with Gefitinib alone, ML-7 alone, or Gefitinib/ML-7 in combination. Cell viability was determined by CCK-8 assay. Combination index (CI) was calculated by using CompuSyn software. **c** Divide cells into four groups, Control group, Gefitinib alone group, ML-7 alone group and Gefitinib/ML-7 in combination group. Colony-formation assay was carried out in those four groups. Representative images of colony formation are shown in the upper panel. The numbers of cells in the indicated groups were shown in the histogram. The bars indicate the mean ± s.d. Statistically significant differences (t-test), **P < 0.01. **d** Wound healing assay was carried out in those four groups. Representative images of migration were shown in the upper panel. The degrees to which the wounds healed was shown in the histogram. The bars indicate the mean ± s.d. Statistically significant differences (t-test), **P < 0.01, Scale bars = 250 μm. **e** Transwell assay was carried out in those four groups. Representative images of invasion were shown in the upper panel. The proportions of invading cells were shown in the histogram. The bars indicate the mean ± s.d. Statistically significant differences (t-test), **P < 0.01, Scale bars = 200 μm. **f** The number of lung metastasis nodules formed by 143B cells in orthotopic osteosarcoma implanted mice was shown in the right panel, the mice were divided into four groups: mice treated with dmso only, mice treated with Gefitinib only, mice treated with ML-7 only and mice treated with Gefitinib plus ML-7. Statistically significant differences (t-test), **P < 0.01. Representative images of lung morphology and lung metastatic nodules in the four groups were shown in the left panel. Scale bars = 250 μm. **g** Tumor weights of the tumors in the four groups were recorded. Statistically significant differences (t-test), **P < 0.05. **h** The expressions of p-EGFR, p-AKT and p-ERK in vitro were measured by western blotting in different groups. **i** The expressions of p-EGFR, p-AKT and p-ERK in tumor xenograft tissues were measured by western blotting

Since MYLK4 promotes the OS progress via the EGFR signal pathway, we attempt to combine the MYLK4 inhibitor with the EGFR inhibitor in order to see whether this combination synergistically inhibits the growth and metastasis of osteosarcoma in vitro and in vivo. First, we observed that the OS cell line 143B and HOS displayed time-dependent and concentration-dependent cell viability suppression with ML-7 or gefitinib treatment (Supplementary Fig. 6A-D). The IC50 values in 143B and HOS cells at 48 h were 50.66 and 51.4 μM for ML-7 and 36.2 and 40 μM for gefitinib, respectively. Combined treatment obviously restrained cell viability compared with that observed with monotreatment of either gefitinib or ML-7 (Fig. 6a and b). We also observed synergy at different combination concentrations, especially at lower concentrations (Fig. 6a and b). Furthermore, the combination of gefitinib and ML-7 exhibited a synergetic repressive effect on cell proliferation, metastasis, and invasion compared with that observed with monotreatment (Fig. 6c-e). Then, we further confirmed this result using orthotopic xenograft models, which were divided into four groups: control group, gefitinib and ML-7 single drug treated group and gefitinib plus ML-7 treated group. HE staining showed fewer pulmonary metastatic nodules in the group treated with Gefitinib plus ML-7 compared with those observed in the monotherapy and control groups (Fig. 6f). As shown in Fig. 6g, the combination treatment contributed to a more obvious anti-tumor effect compared with that in the monotherapy groups, as determined by tumor weight. Western blotting showed that the combination treatment had an increased inhibiting effect on the EGFR signal pathway and the downstream MAPK and AKT signal pathways in vitro and in vivo compared with that observed for monotreatment with either gefitinib or ML-7 (Fig. 6h and i). Therefore, these results demonstrated that the combination therapy of MYLK4 and EGFR inhibitors suppressed growth and metastasis to a greater extent than monotherapy.

## Discussion

In our study, we first comprehensively analyzed the role of MYLK4 in the regulation of growth and metastasis of OS. We discovered that MYLK4 expression was not only higher in OS tissues (compared with normal human osteoblasts) but also showed higher expression in patients with OS and metastases (compared with the non-metastasis group). Furthermore, MYLK4 overexpression promoted the proliferation and metastasis of OS cells. On the contrary, MYLK4 knockdown inhibited their proliferation and metastasis. Moreover, MYLK4 interacted with the EGFR and promoted growth and metastasis through the EGFR signaling pathway. Furthermore, the MYLK4 inhibitor ML-7 and the EGFR inhibitor gefitinib synergize in suppressing OS growth and metastasis in vitro and in vivo.

MYLK, as one member of MYLK family, is important to cell adhesion, which is a process central to metastasis and migration [24]. In hepatocellular carcinoma, Lin et al. showed MYLK had higher expression in human HCC than adjacent liver specimens [25]. Consistent with this notion, we detected the expression of all four members of the MYLK family in the TARGET database and found MYLK4 had high expression in metastatic OS tissues. Furthermore, an independent GEO database, showed that MYLK4 had a higher expression in OS tissues than in normal human osteoblasts. Through the univariate Cox proportional regression analysis, MYLK4 was found to be related to the poor prognosis of patients with OS in the TARGET database.

Prior studies have shown that MYLK can phosphorylate MLC and that suppression of MLC phosphorylation causes failure of cytokinesis and results in multipolarity in cancer cells [26]. MLC is a critical step in facilitating the association between myosin and F-actin, which may generate a contractile force [27]. Recent studies found that cytoskeleton rearrangement had a strong relationship with EMT [28], which has vital roles in the invasion and metastasis of mesenchymal-derived tumors such as OS [29]. Consistently, we found that overexpression of MYLK4 increased the expression of N-cadherin, Twist and Snail and decreased the expression of E-cadherin; this reaction promoted EMT progress, which resulted in increased metastasis of OS cells. Moreover, MYLK4 knockdown decreased the expression of N-cadherin, Twist and Snail and increased the expression of E-cadherin; this restrained the EMT progress, which resulted in decreased metastasis of OS cells. To further demonstrate the role of MYLK4 in OS metastasis, we constructed MYLK4-knockdown orthotopic OS-implanted mice and found knockdown of MYLK4 restrained lung metastasis of OS more than that in the control group. Transforming growth factor-β (TGF-β) signaling is one of the most vital EMT inducers, which is an important process in tumor development, resulting in cancer cell migration and invasion [30]. Consistently, in our study, expression of MYLK4 was up regulated after TGF-β stimulation; however, MYLK4 knockdown retarded EMT progress induced by TGF-β. Targeting myosin light chain kinase with kinase inhibitors might be an effective strategy to inhibit tumor metastasis. Cui et al. showed that ML-7, a kinase inhibitor of myosin light chain kinase, restrained the development of breast cancer cells via the p38 pathway [31]. Moreover, also in breast cancer cells, Zhou et al. found ML-7 restrained proliferation and migration of breast cancer through ERK1/2 signaling pathway [32]. These results were in keeping with our research as we found ML-7 could suppress the proliferation and migration of OS cells in vitro and vivo. Collectively, our results suggest that MYLK4 influences the development of OS.

Cancer cell migration is a complex process wherein multiple signaling molecules participate. Signaling cascades initiated by adhesion molecules or growth factor receptors (such as the EGFR) control cell migration [33]. EGFR is a member of the ErbB family and has important roles in essential cellular functions including proliferation and invasion [15]. Previous studies have shown that the EGFR signal pathway has a vital role in OS progression. Lin et al. demonstrated that physakengose G induced apoptosis of OS cells through EGFR/mTOR signaling [34]. Yuan et al. found that MAT2B promotes proliferation of OS cells by targeting the EGFR signaling [35]. Consistent with this notion, through mass spectrometry analysis, we found EGFR was a new binding protein of MYLK4. Overexpression of MYLK4 promoted EGFR and the downstream signaling pathway. However, MYLK4 knockdown suppressed EGFR and the downstream signaling pathway. Moreover, in OS tissue microarray analysis, the expression of MYLK4 showed a positive correlation with p-EGFR. Furthermore, our results demonstrated that EGFR overexpression could partially reverse the inhibition of OS cell migration and invasion caused by MYLK4 knockdown. Conversely, the EGFR inhibitor gefitinib could partially reverse the promotion of cell migration and invasion by MYLK4 overexpression. Epidermal growth factor (EGF) is the main ligand that specifically binds to the EGFR. Excitation of the EGF/EGFR signaling pathway promotes the proliferation and invasion of cancer cells [36]. In the present study, EGF activated the EGFR signaling pathway in OS and knockdown of MYLK4 could restrain the EGFR signaling pathway induced by EGF. Son et al. showed that enhanced EGFR signaling via upregulated TGF-β signaling promoted cell migration and invasion in human oral squamous cell carcinoma [37]. In diabetic nephropathy, Chen et al. showed that EGFR transactivation was crucial for TGFβ/Smad3 activation and renal fibrosis [38]. In lung adenocarcinoma, Du et al. found that TGF-β-induced factor homeobox 2 mediated the EGFR-RAS-ERK signaling pathway to enhance the stemness of cancer cells [39]. In live cancer, TGF-β could upregulate the expression of EGFR ligands, which transactivated the EGFR pathway, counteracting its pro-apoptotic response [40]. Therefore, TGF-β signaling and EGFR signaling have a cross talk. Therefore, we conclude that MYLK4’s influence on OS progression is dependent on the EGFR signaling pathway.

One of the hallmarks of tumors is the proliferative signaling that is continuously activated. Based on this, the FDA has approved numerous small-molecule kinase inhibitors in clinical practice for the treatment of cancer patients [41]; tyrosine kinase inhibitors were deemed a valid therapeutic means for many cancer types [42]. In recently studies, Maloney et al. showed that gefitinib could inhibit the metastasis of OS through macrophage receptor interacting protein kinase 2 (RIPK2) [43]. Sakurai et al. proved that gefitinib inhibits the growth of prostate cancer and OS through inhibition of cyclin G-associated kinase (GAK) [44]. Consistently, we also found gefitinib could inhibit the growth and metastasis of OS in vitro and vivo. However, a number of studies showed that OS cells had resistance to EGFR inhibitors and the resistance could be overcome by combination with other treatment. Ji et al. demonstrated that combined EGFR/STAT3 inhibition could overcome resistance of EGFR inhibition [45]. In another study, Gvozdenovic et al. found that co-targeting of IGF-IR and EGFR signaling was a useful therapeutic strategy to overcome resistance EGFR inhibitors in OS [46]. Consistently, we found that combined MYLK4 inhibitor ML-7 and EGFR inhibitor gefitinib had a synergistic effect in restraining the proliferation and metastasis of OS cells in vivo and in vitro. Therefore, a combination therapy of MYLK4 and EGFR inhibitors could be a novel treatment strategy for OS. The detailed molecular mechanism deserves further investigation.

## Conclusions

In conclusion, our findings demonstrate that the function of growth and metastasis in OS cells could be regulated by MYLK4 via the EGFR signaling pathway. This is the first study to show that the combination therapy of MYLK4 and EGFR inhibitors may act as a potential therapeutic approach for OS. These data indicated that MYLK4 can be a potential diagnostic and therapeutic target in patients with OS.

## Supplementary Information

**Additional file 1: Figure S1.** Expression of Myosin Light Chain Kinase family members in database and OS cell lines. Expression of MYLK A), MYLK2 B), and MYLK3 C) between non-metastasis and metastasis osteosarcoma samples in TARGET database. Expression of MYLK D), MYLK2 E), MYLK3 F) and MYLK4 G) between normal human osteoblasts and OS tumor samples in GSE12865. H) Expressions of MYLK4 in OS cell lines which were detected by western blotting. Statistically significant differences (t-test), NS, nonsignificant, *P < 0.05.

**Additional file 2: Figure S2.** Induced EMT in OS can be suppressed by knockdown of MYLK4. A) The expression of MYLK4 was detected by western blotting in the induction by TGF-β (10 ng/ml). B) The expression of EMT markers was detected by western blotting in MYLK4-knockdown cells and the control cells induced by TGF-β (10 ng/ml).

**Additional file 3: Figure S3.** Knockdown of MYLK4 expression alters cytoskeleton organization. Knockdown of MYLK4 leads a decrease of F-actin stress fibers and disorganization of F-actin architectures.

**Additional file 4: Figure S4.** MYLK4 interacts with EGFR and phosphorylates EGFR. A) Pan-Tyr of MYLK4 was detected with or without Gefitinib in Flag-MYLK4 143B cells. B) His-tag pull-down assay was performed to investigate the direct interaction between MYLK4 and EGFR. C) An in vitro kinase assay for the detection of EGFR phosphorylation.

**Additional file 5: Figure S5.** The function of MYLK4 is dependent on EGFR signaling. A) The expressions of p-EGFR, p-AKT and p-ERK were detected by western blotting in MYLK4 knockdown and the control cell after co-transfecting with indicated plasmids for 48 h. B) The expression of p-EGFR, p-AKT and p-ERK were detected by western blotting in MYLK4 overexpression cell treated by indicated gefitinib (25 μM) or dmso for 24 h. C) Wound healing assay was carried out in MYLK4 knockdown cell after co-transfecting with indicated plasmids for 48 h. Representative images of migration were shown in the upper panel. The degrees to which the wounds healed was shown in the histogram. The bars indicate the mean ± s.d. Statistically significant differences (t-test), **P < 0.01, Scale bars = 250 μm. D) Transwell assay was carried out in MYLK4 knockdown cell after co-transfecting with indicated plasmids for 48 h. Representative images of invasion were shown in the upper panel. The proportions of invading cells were shown in the histogram. The bars indicate the mean ± s.d. Statistically significant differences (t-test), **P < 0.01, Scale bars = 200 μm. **e** Wound healing assay was carried out in MYLK4 overexpression cells treated by indicated gefitinib (25 μM) or dmso. Representative images of migration were shown in the upper panel. The degrees to which the wounds healed was shown in the histogram. The bars indicate the mean ± s.d. Statistically significant differences (t-test), *P < 0.05, **P < 0.01, Scale bars = 250 μm. **f** Transwell assay was carried out in MYLK4 overexpression cells treated by indicated gefitinib (25 μM) or dmso. Representative images of invasion were shown in the upper panel. The proportions of invading cells were shown in the histogram. The bars indicate the mean ± s.d. Statistically significant differences (t-test), **P < 0.01, Scale bars = 200 μm.

**Additional file 6: Figure S6.** Cell viability assay of ML-7 and Gefitinib in OS cells. 143B cells were treated by different concentration of Gefitinib A) and ML-7 B) for different periods. HOS cells were treated by different concentration of Gefitinib C) and ML-7 D) for different periods.

**Additional file 7: Table S1.** Clinical pathological parameters of patients with OS in TARGET database.

**Additional file 8: Table S2.** Clinical characteristics of the osteosarcoma patients in immunohistochemistry assay.

**Additional file 9: Table S3.** Survival time of the osteosarcoma patients in TARGET database.

**Additional file 10: Table S4.** The 10 osteosarcoma-associated genes in mass spectrometry experiment.

## Data Availability

The datasets analyzed during the current study are not publicly available but are available from the corresponding author on reasonable request.

## Abbreviations

OS: Osteosarcoma
MYLK4: Myosin Light Chain Kinase Family Member 4
EGFR: Epidermal growth factor receptor
IP: immunoprecipitation
EGF: Epidermal growth factor
IHC: Immunohistochemistry
TARGET: Therapeutically Applicable Research to Generate Effective Treatments
