# Supplementary material for: MYLK4 promotes tumor progression through the activation of epidermal growth factor receptor signaling in osteosarcoma
Source: J Exp Clin Cancer Res. 2021 May 12;40:166. doi: 10.1186/s13046-021-01965-z (PMC8114533; doi:10.1186/s13046-021-01965-z)
Supplement: Supplementary file 1 — Additional file 1: Figure S1. Expression of Myosin Light Chain Kinase family members in database and OS cell lines. Expression of MYLK A), MYLK2 B), and MYLK3 C) between non-metastasis and metastasis osteosarcoma samples in TARGET database. Expression of MYLK D), MYLK2 E), MYLK3 F) and MYLK4 G) between normal human osteoblasts and OS tumor samples in GSE12865. H) Expressions of MYLK4 in OS cell lines which were detected by western blotting. Statistically significant differences (t-test), NS, nonsignificant, *P < 0.05. [file 13046_2021_1965_MOESM1_ESM.docx]

**
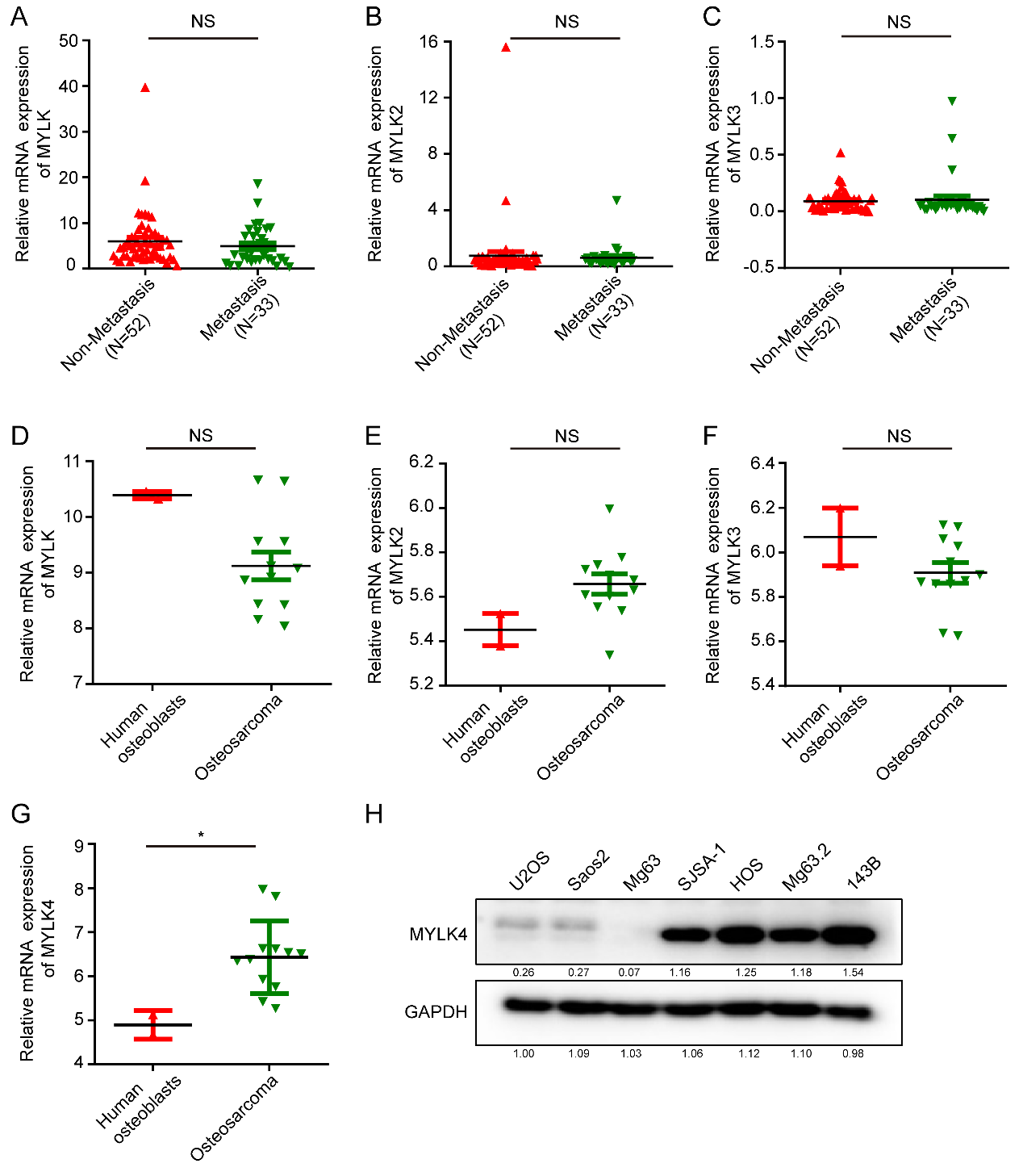
**

**Figure S1.** Expression of Myosin Light Chain Kinase family members in database and OS cell lines. Expression of MYLK A), MYLK2 B), and MYLK3 C) between non-metastasis and metastasis osteosarcoma samples in TARGET database. Expression of MYLK D), MYLK2 E), MYLK3 F) and MYLK4 G) between normal human osteoblasts and OS tumor samples in GSE12865. H) Expressions of MYLK4 in OS cell lines which were detected by western blotting. Statistically significant differences (t-test), NS, nonsignificant, *P<0.05.
