# Supplementary material for: MYLK4 promotes tumor progression through the activation of epidermal growth factor receptor signaling in osteosarcoma
Source: J Exp Clin Cancer Res. 2021 May 12;40:166. doi: 10.1186/s13046-021-01965-z (PMC8114533; doi:10.1186/s13046-021-01965-z)
Supplement: Supplementary file 2 — Additional file 2: Figure S2. Induced EMT in OS can be suppressed by knockdown of MYLK4. A) The expression of MYLK4 was detected by western blotting in the induction by TGF-β (10 ng/ml). B) The expression of EMT markers was detected by western blotting in MYLK4-knockdown cells and the control cells induced by TGF-β (10 ng/ml). [file 13046_2021_1965_MOESM2_ESM.docx]

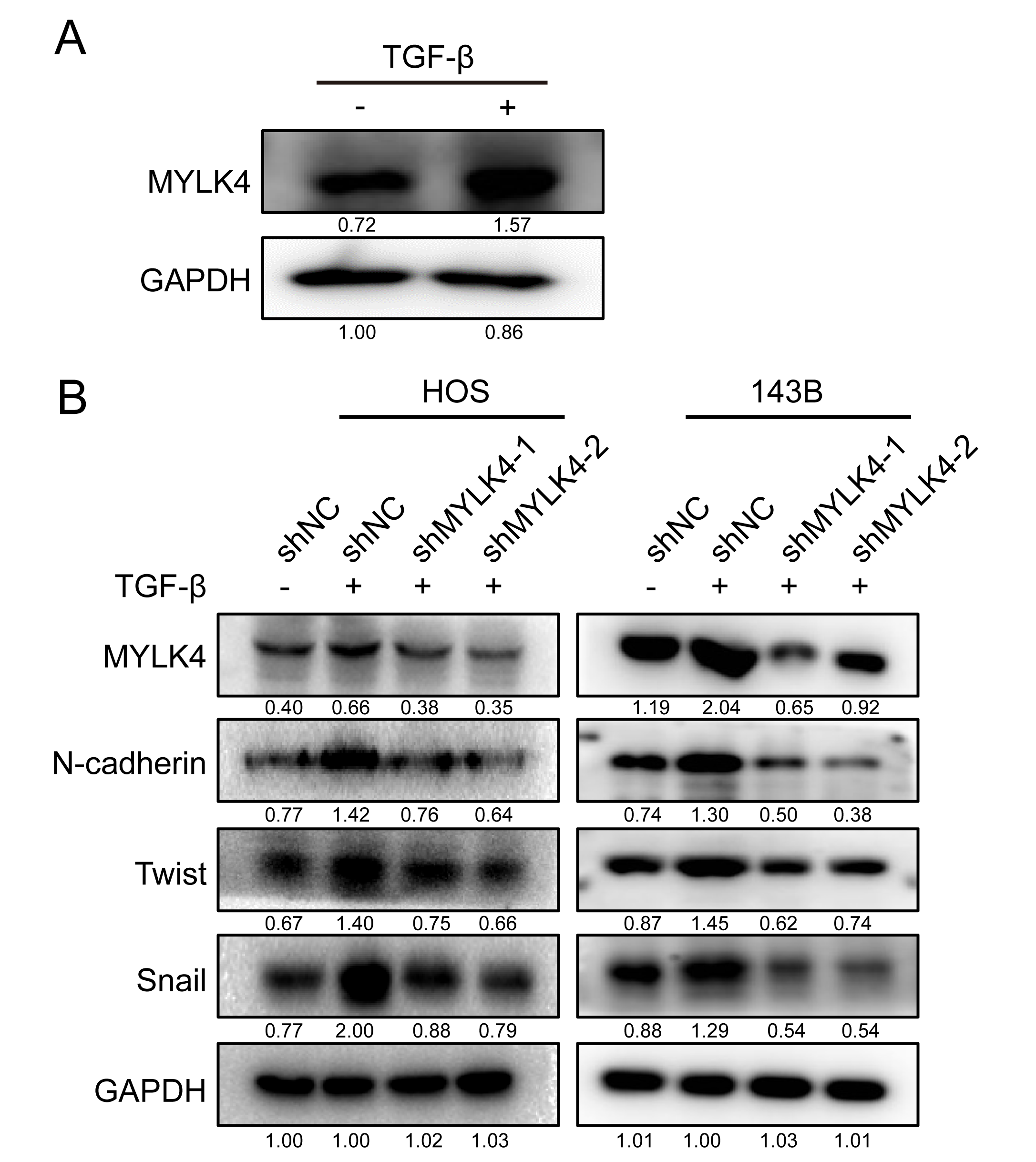


**Figure S2.** Induced EMT in OS can be suppressed by knockdown of MYLK4. A) The expression of MYLK4 was detected by western blotting in the induction by TGF-β (10 ng/ml). B) The expression of EMT markers was detected by western blotting in MYLK4-knockdown cells and the control cells induced by TGF-β (10 ng/ml).
