# Supplementary material for: MYLK4 promotes tumor progression through the activation of epidermal growth factor receptor signaling in osteosarcoma
Source: J Exp Clin Cancer Res. 2021 May 12;40:166. doi: 10.1186/s13046-021-01965-z (PMC8114533; doi:10.1186/s13046-021-01965-z)
Supplement: Supplementary file 3 — Additional file 3: Figure S3. Knockdown of MYLK4 expression alters cytoskeleton organization. Knockdown of MYLK4 leads a decrease of F-actin stress fibers and disorganization of F-actin architectures. [file 13046_2021_1965_MOESM3_ESM.docx]

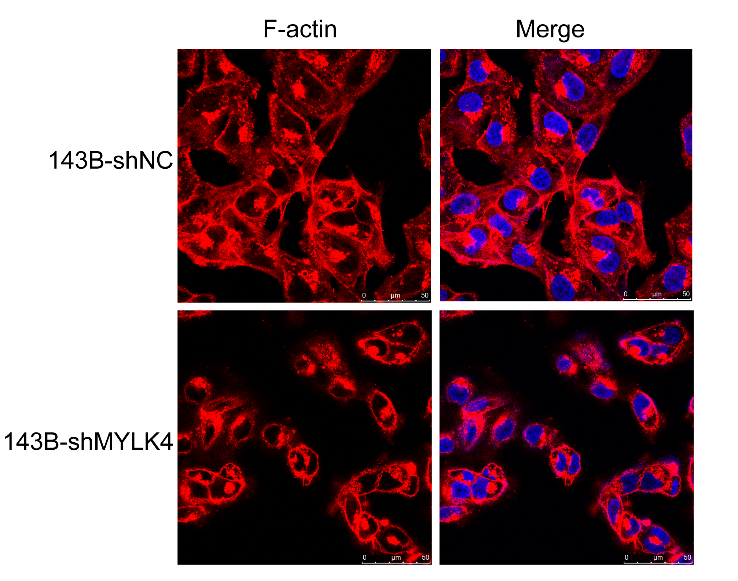


**Figure S3**. Knockdown of MYLK4 expression alters cytoskeleton organization. Knockdown of MYLK4 leads a decrease of F-actin stress fibers and disorganization of F-actin architectures.
