# Supplementary material for: MYLK4 promotes tumor progression through the activation of epidermal growth factor receptor signaling in osteosarcoma
Source: J Exp Clin Cancer Res. 2021 May 12;40:166. doi: 10.1186/s13046-021-01965-z (PMC8114533; doi:10.1186/s13046-021-01965-z)
Supplement: Supplementary file 4 — Additional file 4: Figure S4. MYLK4 interacts with EGFR and phosphorylates EGFR. A) Pan-Tyr of MYLK4 was detected with or without Gefitinib in Flag-MYLK4 143B cells. B) His-tag pull-down assay was performed to investigate the direct interaction between MYLK4 and EGFR. C) An in vitro kinase assay for the detection of EGFR phosphorylation. [file 13046_2021_1965_MOESM4_ESM.docx]

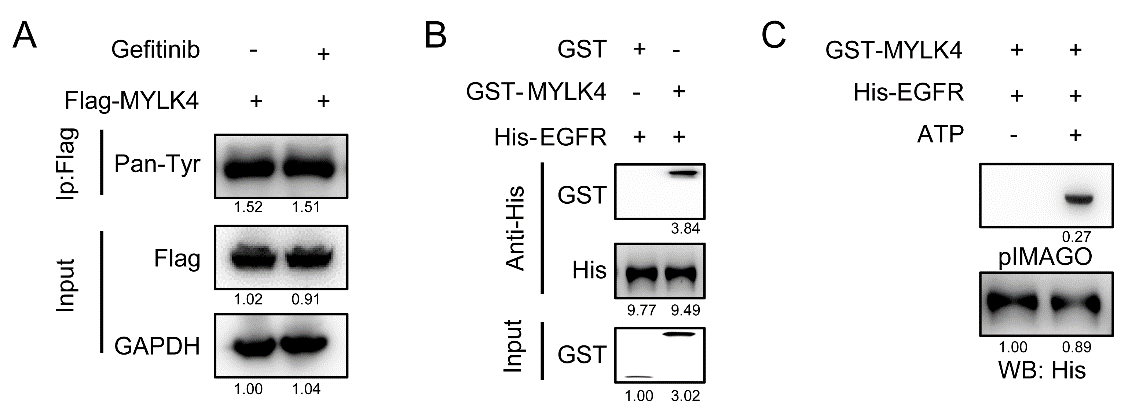


**Figure S4.** MYLK4 interacts with EGFR and phosphorylates EGFR. A) Pan-Tyr of MYLK4 was detected with or without Gefitinib in Flag-MYLK4 143B cells. B) His-tag pull-down assay was performed to investigate the direct interaction between MYLK4 and EGFR. C) An in vitro kinase assay for the detection of EGFR phosphorylation.
