# Supplementary material for: MYLK4 promotes tumor progression through the activation of epidermal growth factor receptor signaling in osteosarcoma
Source: J Exp Clin Cancer Res. 2021 May 12;40:166. doi: 10.1186/s13046-021-01965-z (PMC8114533; doi:10.1186/s13046-021-01965-z)
Supplement: Supplementary file 5 — Additional file 5: Figure S5. The function of MYLK4 is dependent on EGFR signaling. A) The expressions of p-EGFR, p-AKT and p-ERK were detected by western blotting in MYLK4 knockdown and the control cell after co-transfecting with indicated plasmids for 48 h. B) The expression of p-EGFR, p-AKT and p-ERK were detected by western blotting in MYLK4 overexpression cell treated by indicated gefitinib (25 μM) or dmso for 24 h. C) Wound healing assay was carried out in MYLK4 knockdown cell after co-transfecting with indicated plasmids for 48 h. Representative images of migration were shown in the upper panel. The degrees to which the wounds healed was shown in the histogram. The bars indicate the mean ± s.d. Statistically significant differences (t-test), **P < 0.01, Scale bars = 250 μm. D) Transwell assay was carried out in MYLK4 knockdown cell after co-transfecting with indicated plasmids for 48 h. Representative images of invasion were shown in the upper panel. The proportions of invading cells were shown in the histogram. The bars indicate the mean ± s.d. Statistically significant differences (t-test), **P < 0.01, Scale bars = 200 μm. e Wound healing assay was carried out in MYLK4 overexpression cells treated by indicated gefitinib (25 μM) or dmso. Representative images of migration were shown in the upper panel. The degrees to which the wounds healed was shown in the histogram. The bars indicate the mean ± s.d. Statistically significant differences (t-test), *P < 0.05, **P < 0.01, Scale bars = 250 μm. f Transwell assay was carried out in MYLK4 overexpression cells treated by indicated gefitinib (25 μM) or dmso. Representative images of invasion were shown in the upper panel. The proportions of invading cells were shown in the histogram. The bars indicate the mean ± s.d. Statistically significant differences (t-test), **P < 0.01, Scale bars = 200 μm. [file 13046_2021_1965_MOESM5_ESM.docx]

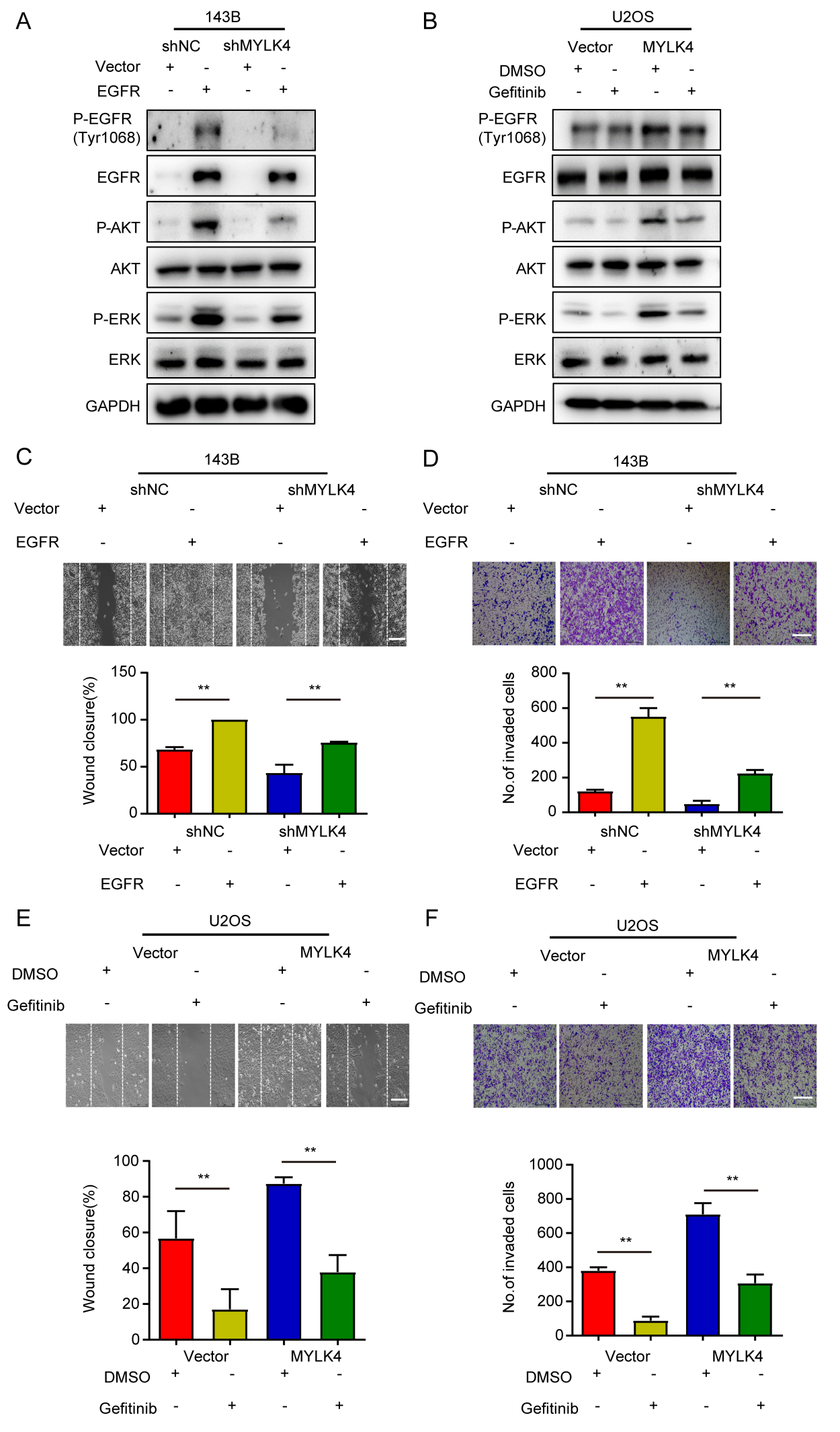


**Figure S5**. The function of MYLK4 is dependent on EGFR signaling. A) The expressions of p-EGFR, p-AKT and p-ERK were detected by western blotting in MYLK4 knockdown and the control cell after co-transfecting with indicated plasmids for 48h. B) The expression of p-EGFR, p-AKT and p-ERK were detected by western blotting in MYLK4 overexpression cell treated by indicated gefitinib (25 μM) or dmso for 24h. C) Wound healing assay was carried out in MYLK4 knockdown cell after co-transfecting with indicated plasmids for 48h. Representative images of migration were shown in the upper panel. The degrees to which the wounds healed was shown in the histogram. The bars indicate the mean±s.d. Statistically significant differences (t-test), **P<0.01, Scale bars = 250μm. D) Transwell assay was carried out in MYLK4 knockdown cell after co-transfecting with indicated plasmids for 48 h. Representative images of invasion were shown in the upper panel. The proportions of invading cells were shown in the histogram. The bars indicate the mean±s.d. Statistically significant differences (t-test), **P<0.01, Scale bars = 200 μm. E) Wound healing assay was carried out in MYLK4 overexpression cells treated by indicated gefitinib (25 μM) or dmso. Representative images of migration were shown in the upper panel. The degrees to which the wounds healed was shown in the histogram. The bars indicate the mean±s.d. Statistically significant differences (t-test), *P<0.05, **P<0.01, Scale bars = 250μm. F) Transwell assay was carried out in MYLK4 overexpression cells treated by indicated gefitinib (25 μM) or dmso. Representative images of invasion were shown in the upper panel. The proportions of invading cells were shown in the histogram. The bars indicate the mean±s.d. Statistically significant differences (t-test), **P<0.01, Scale bars = 200 μm.
