# Supplementary material for: MYLK4 promotes tumor progression through the activation of epidermal growth factor receptor signaling in osteosarcoma
Source: J Exp Clin Cancer Res. 2021 May 12;40:166. doi: 10.1186/s13046-021-01965-z (PMC8114533; doi:10.1186/s13046-021-01965-z)
Supplement: Supplementary file 6 — Additional file 6: Figure S6. Cell viability assay of ML-7 and Gefitinib in OS cells. 143B cells were treated by different concentration of Gefitinib A) and ML-7 B) for different periods. HOS cells were treated by different concentration of Gefitinib C) and ML-7 D) for different periods. [file 13046_2021_1965_MOESM6_ESM.docx]

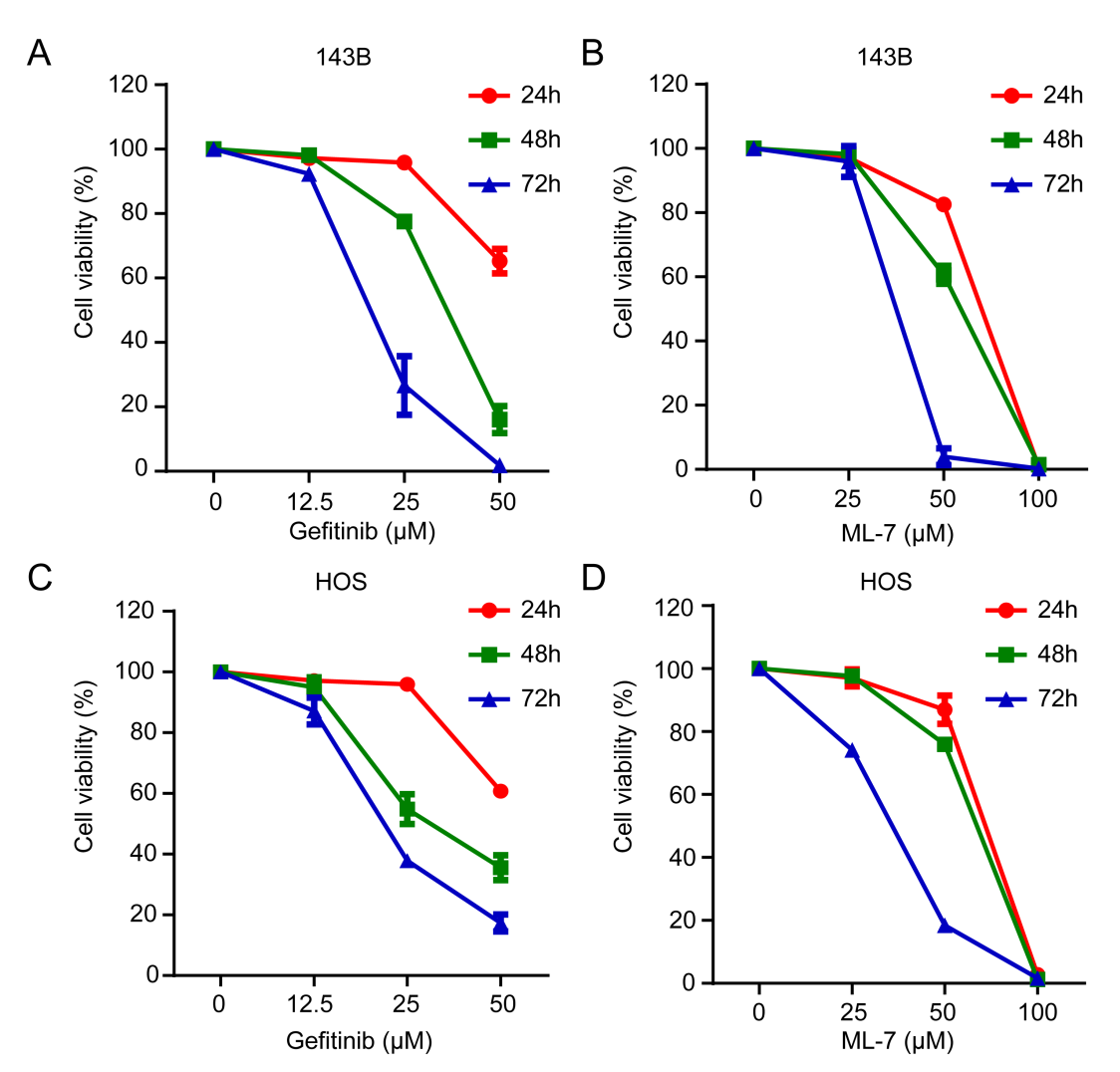


**Figure S6.** Cell viability assay of ML-7 and Gefitinib in OS cells. 143B cells were treated by different concentration of Gefitinib A) and ML-7 B) for different periods. HOS cells were treated by different concentration of Gefitinib C) and ML-7 D) for different periods.
