# Supplementary material for: MYLK4 promotes tumor progression through the activation of epidermal growth factor receptor signaling in osteosarcoma
Source: J Exp Clin Cancer Res. 2021 May 12;40:166. doi: 10.1186/s13046-021-01965-z (PMC8114533; doi:10.1186/s13046-021-01965-z)
Supplement: Supplementary file 7 — Additional file 7: Table S1. Clinical pathological parameters of patients with OS in TARGET database. [file 13046_2021_1965_MOESM7_ESM.docx]

**Table S1.** Clinical pathological parameters of patients with OS in TARGET database

| Characteristic | TARGET (with intact survival time)(n=85) |
| --- | --- |
| Age (years) |  |
| <=18 | 66 |
| >18 | 19 |
| Survival status |  |
| Alive | 56 |
| Dead | 29 |
| Gender |  |
| Female | 37 |
| Male | 48 |
| Metastatic |  |
| Metastatic | 33 |
| Non-metastatic | 52 |
