# Supplementary material for: MYLK4 promotes tumor progression through the activation of epidermal growth factor receptor signaling in osteosarcoma
Source: J Exp Clin Cancer Res. 2021 May 12;40:166. doi: 10.1186/s13046-021-01965-z (PMC8114533; doi:10.1186/s13046-021-01965-z)
Supplement: Supplementary file 8 — Additional file 8: Table S2. Clinical characteristics of the osteosarcoma patients in immunohistochemistry assay. [file 13046_2021_1965_MOESM8_ESM.docx]

**Table S2.** Clinical characteristics of the osteosarcoma patients in immunohistochemistry assay

| Characteristic | n=16 |
| --- | --- |
| Gender |  |
| Male | 8 (50%) |
| Female | 8 (50%) |
| Tumor metastasis |  |
| Non-metastasis | 8 (50%) |
| Metastasis | 8 (50%) |
| Primary site |  |
| Tibia | 5 (31.2%) |
| Femur | 7 (43.8%) |
| Wrist | 1 (6.3%) |
| Humerus | 1 (6.3%) |
| Pelvis | 1 (6.3%) |
| Ilium | 1 (6.3%) |
