# Supplementary material for: MYLK4 promotes tumor progression through the activation of epidermal growth factor receptor signaling in osteosarcoma
Source: J Exp Clin Cancer Res. 2021 May 12;40:166. doi: 10.1186/s13046-021-01965-z (PMC8114533; doi:10.1186/s13046-021-01965-z)
Supplement: Supplementary file 9 — Additional file 9: Table S3. Survival time of the osteosarcoma patients in TARGET database. [file 13046_2021_1965_MOESM9_ESM.docx]

**Table S3.** Survival time of the osteosarcoma patients in TARGET database

| Id  (n=85) | Survival（0=survival, 1=death） | Overall Survival Time in Days | Event Free Survival Time in  Days |
| --- | --- | --- | --- |
| TARGET-40-0A4HY5 | 1 | 290 | 218 |
| TARGET-40-0A4I0Q | 1 | 519 | 519 |
| TARGET-40-0A4I42 | 1 | 619 | 351 |
| TARGET-40-0A4I4O | 1 | 627 | 235 |
| TARGET-40-0A4I6O | 1 | 1906 | 484 |
| TARGET-40-PAKZZK | 1 | 1515 | 452 |
| TARGET-40-PALECC | 1 | 754 | 384 |
| TARGET-40-PALFYN | 1 | 1003 | 651 |
| TARGET-40-PALZGU | 1 | 2882 | 1452 |
| TARGET-40-PAMEKS | 1 | 857 | 385 |
| TARGET-40-PAMJXS | 1 | 3862 | 3184 |
| TARGET-40-PAMLKS | 1 | 180 | 71 |
| TARGET-40-PAMRHD | 1 | 74 | 39 |
| TARGET-40-PAMYYJ | 1 | 271 | 128 |
| TARGET-40-PANMIG | 1 | 776 | 513 |
| TARGET-40-PANPUM | 1 | 295 | 46 |
| TARGET-40-PAPKWD | 1 | 422 | 192 |
| TARGET-40-PAPNVD | 1 | 922 | 594 |
| TARGET-40-PARDAX | 1 | 679 | 105 |
| TARGET-40-PARFTG | 1 | 653 | 53 |
| TARGET-40-PARJXU | 1 | 1631 | 811 |
| TARGET-40-PARKAF | 1 | 1061 | 56 |
| TARGET-40-PATKSS | 1 | 758 | 63 |
| TARGET-40-PATMPU | 1 | 1579 | 498 |
| TARGET-40-PATMXR | 1 | 386 | 68 |
| TARGET-40-PATUXZ | 1 | 510 | 510 |
| TARGET-40-PAUTYB | 1 | 537 | 527 |
| TARGET-40-PAUVUL | 1 | 347 | 237 |
| TARGET-40-PAUXPZ | 1 | 606 | 419 |
| TARGET-40-0A4HLD | 0 | 3946 | 3946 |
| TARGET-40-0A4HMC | 0 | 377 | 377 |
| TARGET-40-0A4HX8 | 0 | 1750 | 3050 |
| TARGET-40-0A4HXS | 0 | 2948 | 2948 |
| TARGET-40-0A4I0S | 0 | 603 | 603 |
| TARGET-40-0A4I0W | 0 | 683 | 683 |
| TARGET-40-0A4I3S | 0 | 1844 | 1844 |
| TARGET-40-0A4I48 | 0 | 1616 | 1616 |
| TARGET-40-0A4I4M | 0 | 708 | 708 |
| TARGET-40-0A4I5B | 0 | 619 | 619 |
| TARGET-40-0A4I65 | 0 | 5840 | 1993 |
| TARGET-40-0A4I8U | 0 | 983 | 983 |
| TARGET-40-0A4I9K | 0 | 2257 | 2257 |
| TARGET-40-PAKFVX | 0 | 922 | 361 |
| TARGET-40-PAKXLD | 0 | 2462 | 578 |
| TARGET-40-PALHRL | 0 | 4374 | 4374 |
| TARGET-40-PALKDP | 0 | 3282 | 3282 |
| TARGET-40-PALKGN | 0 | 2122 | 2122 |
| TARGET-40-PALWWX | 0 | 2163 | 2163 |
| TARGET-40-PAMHLF | 0 | 1913 | 1913 |
| TARGET-40-PAMHYN | 0 | 3900 | 3900 |
| TARGET-40-PAMTCM | 0 | 3002 | 3002 |
| TARGET-40-PANGPE | 0 | 3378 | 833 |
| TARGET-40-PANGRW | 0 | 3378 | 3378 |
| TARGET-40-PANSEN | 0 | 2256 | 2256 |
| TARGET-40-PANVJJ | 0 | 3095 | 3095 |
| TARGET-40-PANXSC | 0 | 1217 | 366 |
| TARGET-40-PANZHX | 0 | 1323 | 812 |
| TARGET-40-PANZZJ | 0 | 1538 | 1538 |
| TARGET-40-PAPIJR | 0 | 1719 | 1719 |
| TARGET-40-PAPWWC | 0 | 2520 | 1933 |
| TARGET-40-PAPXGT | 0 | 2526 | 2526 |
| TARGET-40-PARBGW | 0 | 286 | 286 |
| TARGET-40-PARGTM | 0 | 2610 | 2610 |
| TARGET-40-PASEBY | 0 | 2049 | 42 |
| TARGET-40-PASEFS | 0 | 2112 | 2112 |
| TARGET-40-PASFCV | 0 | 2062 | 2062 |
| TARGET-40-PASKZZ | 0 | 542 | 542 |
| TARGET-40-PASNZV | 0 | 1747 | 1747 |
| TARGET-40-PASRNE | 0 | 1707 | 512 |
| TARGET-40-PASSLM | 0 | 1865 | 1865 |
| TARGET-40-PASUUH | 0 | 1625 | 1625 |
| TARGET-40-PASYUK | 0 | 1870 | 1870 |
| TARGET-40-PATAWV | 0 | 1702 | 1702 |
| TARGET-40-PATEEM | 0 | 1451 | 961 |
| TARGET-40-PATJVI | 0 | 1575 | 1575 |
| TARGET-40-PATMIF | 0 | 1580 | 1580 |
| TARGET-40-PATPBS | 0 | 1468 | 1468 |
| TARGET-40-PAUBIT | 0 | 1029 | 1029 |
| TARGET-40-PAUTWB | 0 | 687 | 598 |
| TARGET-40-PAUUML | 0 | 672 | 672 |
| TARGET-40-PAUYTT | 0 | 579 | 579 |
| TARGET-40-PAVALD | 0 | 551 | 551 |
| TARGET-40-PAVCLP | 0 | 511 | 511 |
| TARGET-40-PAVDTY | 0 | 369 | 369 |
| TARGET-40-PAVECB | 0 | 476 | 476 |
