# Supplementary material for: MYLK4 promotes tumor progression through the activation of epidermal growth factor receptor signaling in osteosarcoma
Source: J Exp Clin Cancer Res. 2021 May 12;40:166. doi: 10.1186/s13046-021-01965-z (PMC8114533; doi:10.1186/s13046-021-01965-z)
Supplement: Supplementary file 10 — Additional file 10: Table S4. The 10 osteosarcoma-associated genes in mass spectrometry experiment. [file 13046_2021_1965_MOESM10_ESM.docx]

**Table S4.** The 10 osteosarcoma-associated genes in mass spectrometry experiment

| Protein name | prot_score |
| --- | --- |
| HSP90AA1 | 5284 |
| MYLK4 | 2883 |
| CDK1 | 388 |
| RHOA | 304 |
| ITGB1 | 152 |
| MAPK1 | 136 |
| RAC1 | 109 |
| EGFR | 82 |
| CDC5L | 42 |
| EP300 | 37 |
| PTK2 | 34 |
